# Isolation and Characterisation of Mesenchymal Stem Cells from Rat Bone Marrow and the Endosteal Niche: A Comparative Study

**DOI:** 10.1155/2018/6869128

**Published:** 2018-03-22

**Authors:** Norhayati Yusop, Paul Battersby, Amr Alraies, Alastair J. Sloan, Ryan Moseley, Rachel J. Waddington

**Affiliations:** ^1^Oral and Biomedical Sciences, School of Dentistry and Cardiff Institute Tissue Engineering and Repair, Cardiff University, Cardiff, UK; ^2^School of Dental Sciences, University Sains Malaysia, Kelantan, Malaysia

## Abstract

Within bone, mesenchymal stromal cells (MSCs) exist within the bone marrow stroma (BM-MSC) and the endosteal niche, as cells lining compact bone (CB-MSCs). This study isolated and characterised heterogeneous MSC populations from each niche and subsequently investigated the effects of extensive cell expansion, analysing population doublings (PDs)/cellular senescence, colony-forming efficiencies (CFEs), MSC cell marker expression, and osteogenic/adipogenic differentiation. CB-MSCs and BM-MSCs demonstrated similar morphologies and PDs, reaching 100 PDs. Both populations exhibited consistent telomere lengths (12–17 kb), minimal senescence, and positive telomerase expression. CB-MSCs (PD15) had significantly lower CFEs than PD50. CB-MSCs and BM-MSCs both expressed MSC (CD73/CD90/CD105); embryonic (Nanog) and osteogenic markers (Runx2, osteocalcin) but no hematopoietic markers (CD45). CB-MSCs (PD15) strongly expressed Oct4 and p16^INK4A^. At early PDs, CB-MSCs possessed a strong osteogenic potency and low potency for adipogenesis, whilst BM-MSCs possessed greater overall bipotentiality for osteogenesis and adipogenesis. At PD50, CB-MSCs demonstrated reduced potency for both osteogenesis and adipogenesis, compared to BM-MSCs at equivalent PDs. This study demonstrates similarities in proliferative and mesenchymal cell characteristics between CB-MSCs and BM-MSCs, but contrasting multipotentiality. Such findings support further comparisons of human CB-MSCs and BM-MSCs, facilitating selection of optimal MSC populations for regenerative medicine purposes.

## 1. Introduction

The bone marrow cavity contains a rich source of mesenchymal stromal cells (BM-MSCs). These MSCs can be considered as a distinct type of stromal progenitor cells with defined capabilities for self-renewal and differentiation into lineages of mesenchymal origins, such as bone, fat, and various other collagenous connective tissues [1, 2]. Consequently, BM-MSCs are highly considered to offer great potential for application in stem cell repair and regenerative therapies [3], most notably for bone itself. They are also often used in the development of *in vitro* models of disease progression and for the monitoring of therapeutic efficiency in accelerating a wide variety of clinical outcomes [4–7].

BM-MSCs have been described within two distinct niches within the bone environment, namely, the perivascular niche organized around sinusoidal endothelial cells and the endosteal niche centralized around preosteoblasts and osteoblasts of the bone-lining cells [8]. Via cell-cell contacts, the BM-MSCs of both niches provide a role in supporting the activities of the hematopoietic cells, in addition to facilitating bone remodeling and repair, whether for stress-induced microfractures or major trauma-induced fractures [8]. Furthermore, within both these niches, isolated MSCs represent heterogeneous populations, commonly forming the progenitor cells of adipocytes and osteoblasts where balanced differentiation in favour of osteogenesis is important for successful bone repair. Indeed, dysregulation towards adipogenesis during bone remodeling has been linked to several pathologies of weakened bone seen in obesity, osteopenia, and osteoporosis [9]. Clonal analyses of BM-MSCs have identified cell populations that are described as highly proliferative transit-amplifying cells, capable of forming colonies, and possessing multipotency, alongside cell populations with lower colony-forming efficiencies that are more restricted in their lineage potential [10, 11]. Such understanding has consequences for the protocols used to isolate BM-MSCs, whether for use in cell-based therapies or *in vitro* cell models, where cell populations with defined characteristics are desirable. For most isolations of MSCs from tissues such as the bone, the cell population can be regarded as heterogeneous containing “immature” highly proliferative multipotential cells, along with lineage-committed and differentiated cells with slower proliferative capacity which can vary greatly between sampled individuals [3, 12]. Following isolation, cells are invariably expanded *in vitro* to obtain sufficient numbers. This can lead to further change in the heterogeneous profile of the MSCs, which are highly likely to impact on a whole range of cellular behaviour, such as multipotency, efficacy of differentiation, proliferation, migration, and immunosuppression [12]. However, despite the huge variations in isolation procedures being identified to be a major hindrance to clinical translation, very few studies have compared isolation techniques and the characterisation of the cell populations following expansion.

Classically, MSCs were isolated from the bone marrow tissues of human and rodent species, by manipulating their distinct ability to expand in culture following adherence to plastic culture surfaces, as the techniques have shown a potential in reducing the coculture of hematopoietic cells [13]. However, the property of plastic adherence itself is not sufficient for the isolation of MSCs due to the abundant existence of unwanted hematopoietic cells, endothelial cells, and granulomonocytic cells reported in early and later stages of subculture. Frequently, bone marrow stromal cells are subjected to fractionation on a density gradient solution, such as Ficoll™, to improve the purification strategies, followed by low-density plating methods [14, 15]. Extending from initial studies isolating stem cell populations from the epidermis [16], recent studies have also been successful in the isolation of heterogeneous MSC populations from bone marrow, dental pulp, and the oral mucosa, by virtue that these adult stem cells exhibit high surface *α*_5_*β*_1_ integrin expression, to promote rapid adhesion to fibronectin-coated culture plates, providing easier selection from hematopoietic and endothelial cell types present in this tissue [17–20]. With the growing development of technologies in bone engraftment and skeletal tissue repair, the search for potential MSC candidates with specific pro-osteoinductive and pro-osteoconductive capacities directly derived from endosteal niche has been described as promising for the application in bone repair treatments. Against this clinical aim, recent studies have successfully performed the isolation of MSCs from mouse compact bone explants, suggesting the potential application of this method as an alternative in providing MSCs for bone repair therapies and for *in vitro* models of cellular behaviour [21–23].

This study presents data comparing the isolation, expansion, and characterisation of heterogeneous rat MSCs from bone marrow and compact bone explants. Attention is given to compare the efficiency of each purification method by analyzing cell proliferative capacity, maintenance of mesenchymal stromal/stem cell characteristics, and differentiation potential towards osteogenic and adipogenic lineages, following extensive culture *in vitro*. The successful isolation, expansion, and characterisation of these MSCs revealed that both bone marrow and compact bone offer the potential for two contrasting *in vitro* cell models that differ with respect to their differentiation potency. The results presented thus offer an initial characterisation relating to the differing heterogeneous nature of the MSC populations from each niche, which is valuable in considering appropriateness of the isolation and expansion protocols during study design and provide an initial viability assessment of the potential use of each stromal cell population for future stem cell therapy development.

## 2. Materials and Methods

Bone femurs were harvested from 28-day-old male Wistar rats, sacrificed in accordance with Code of Practice for the Humane Killing of Animals, under Schedule 1 of the Animals (Scientific Procedures) Act, 1986. Prior to dissection, animals were sterilized by immersion in 70% alcohol.

### 2.1. Isolation and Culture of BM-MSCs from Bone Marrow

Six-well plates were coated with 1 ml/well of 10 *μ*g/ml human plasma fibronectin (Sigma-Aldrich, Poole, UK), reconstituted in +PBS (phosphate-buffered saline, supplemented with 1 mM Ca^2+^ and 1 mM Mg^2+^), and left for 24 h at 4°C. The femur and humerus were aseptically dissected, cleaned of all connective tissues, and collected on ice in 10 ml isolation medium (IM), *α*MEM with ribonucleosides and deoxyribonucleosides (ThermoFisher Scientific, Paisley, UK), supplemented with 10% antibiotic-antimycotic (Sigma-Aldrich). Bone marrow cells were collected by flushing of each long bone with 10 ml of complete culture medium (CCM), containing *α*MEM with 20% heat-inactivated foetal bovine serum (FBS, ThermoFisher Scientific), 1% antibiotic-antimycotic, and 100 *μ*m L-ascorbic acid 2-phosphate (Sigma-Aldrich), and filtered through a 70 *μ*m nylon cell strainer into T-75 tissue culture flasks (Greiner Bio-One International, Kremsmünster, Austria). Cells were incubated at 37°C, 5% CO_2_, and nonadherent cells were removed by change of medium after 24 h. On day 3, cells were retrieved using StemPro**®** Accutase**®** (ThermoFisher Scientific) and counted. To preferentially select immature MSCs expressing high levels of cell surface *β*1 integrins, cells were seeded at 4000 cells/cm^2^ in 1 ml of serum-free CCM into each well of fibronectin-coated plates, immediately after removing PBS. After 20 min incubation at 37°C, nonadherent cells were removed by washing with PBS. Adherent cells were cultured in CCM at 37°C, 5% CO_2_. Culture medium was changed every 2-3 days.

### 2.2. Isolation and Culture of Rat Bone Chip Explant CB-MSCs

From the long bone remnants recovered from above, the diaphysis was cut into 1–3 mm^2^ bone chips and digested with gentle agitation in 3 mg/ml collagenase II (Sigma-Aldrich) in *α*MEM for 2 h at 37°C. Digestion medium and released cells were removed, and the bone chips were washed three times with 5 ml CCM. The digested bone chips were then incubated with CCM at 37°C, 5% CO_2_. On day 3, the bone chips and nonadherent cells were discarded by washing with PBS and remaining adherent cells were cultured to allow for colony formation and expansion. After 12 days, CB-MSCs were harvested using StemPro Accutase (Passage 0), counted, and reseeded at 4000 cells/cm^2^ in T-25 culture flasks (Greiner Bio-One International). Culture medium was changed every 2-3 days.

### 2.3. Histological Examination

During the isolation procedure, tissue samples of the bone were taken before flushing away the bone marrow tissue and of the resultant bone chips before and after collagenase treatment. Samples were demineralised in 10% formic acid, for 72 h, and then dehydrated through an ascending concentration of alcohols, which was cleared with xylene prior to embedding in paraffin wax. Five *μ*m sections were cut, mounted onto poly-L-lysine-coated glass slides (ThermoFisher Scientific), dried overnight at 60°C, and then stained with haematoxylin and eosin. Stained sections were mounted using DPX glue (Raymond A Lamb, East Sussex, UK), prior to viewing using a light microscope, with digital images captured.

### 2.4. Assessment of Population Doubling Levels

Upon reaching 70–80% confluency, cells were passaged and cell counts were determined. Population doubling (PD) values were assessed as a proportion of the original number of cells seeded using the formula:

(1) $$\begin{array}{c} \frac{\log10\;\left(cells\;harvested\right)-\log10\;\left(cells\;reseeded\right)}{\log10\;\left(2\right)}. \end{array}$$

Cumulative PD was plotted against time in culture and performed in duplicate for each isolation procedure.

### 2.5. Cell Morphological Analysis

Cellular morphology was regularly examined over the entire culture duration by light microscopy. In addition, cells at PD15, PD50, and PD100 were cultured at a density of 4000 cells/cm^2^ for 24 h in glass chamber slides (BD Biosciences, Oxford, UK) before fixation with 4% (*v*/*v*) paraformaldehyde solution and permeabilisation with 200 *μ*l/well 0.1% Triton-X100 (Sigma-Aldrich), for 30 min. Cells were blocked with 200 *μ*l/well 1% bovine serum albumin (BSA, ThermoFisher Scientific) in Tris-buffered saline (1% BSA-TBS), for 1 h at room temperature, and the actin cytoskeleton was stained with 20 *μ*g ml/ml phalloidin-FITC (Sigma-Aldrich), 1% BSA-TBS and incubated for 1 h at 4°C in darkness. Cells on the glass slides were mounted using Vectashield**®** mounting medium with DAPI (Vector Laboratories Ltd., Peterborough, UK), and images were captured using an ultraviolet (UV) microscope.

### 2.6. Colony-Forming Efficiency

Cells were seeded at a density of 100 cells/well of a 6-well plate and cultured for a period of 12 days. Visual observations were performed every 24 h where colonies of 32 cells or more were recorded.

### 2.7. SA-*β*-Galactosidase Staining for Cell Senescence

Cell senescence was analysed by the presence of senescence-associated- (SA-) *β*-galactosidase staining using a Senescence Cells Histochemical Staining Kit (Sigma-Aldrich), according to manufacturer's instruction. Cells were viewed by light microscopy, and the average percentage blue-stained positive cells were calculated from 5 random fields of view (30 *μ*m^2^).

### 2.8. Telomere Length Determination

Cells were cultured at a density of 1 × 10^4^ cells/cm^2^ to 80% confluence, before treatment with proteinase K, and purified with DNEasy Blood and Tissue Kit (Qiagen, Crawley, UK). Purified DNA was run on a 0.8% (*w*/*v*) agarose gels and Southern blotted onto nylon membranes according to manufacturer's instruction for the TeloTAGGG Telomere Length Assay kit (Roche, Welwyn Garden City, UK).

### 2.9. Reverse Transcription PCR

Total RNA was extracted using the RNeasy® Mini kit and QIAShredder (Qiagen Ltd., Crawley, UK). RNA purity and quantity were determined at 260 nm/280 nm absorbance. cDNA was synthesised from 500 ng of total RNA using 5 *μ*l 5X Moloney murine leukaemia virus (M-MLV) buffer, 0.5 *μ*g random primers, 0.625 *μ*l RNasin, 1.25 *μ*l deoxynucleotide triphosphates (dNTPs; 10 mM), and 1 *μ*l M-MLV reverse transcriptase, reconstituted to 15 *μ*l with DNase-free water (all Promega, Southampton, UK, total volume 25 *μ*l), at 37°C for 1 h, followed by 95°C for 5 min.

cDNA was synthesized from 500 ng of total RNA using 1 *μ*l random primer (Promega), added to RNAase-free water to a final volume of 15 *μ*l. The sample was run at 70°C for 5 min. RT reaction mix was prepared, containing 5 *μ*l 5× Moloney murine leukemia virus reverse transcriptase (MMLV) reaction buffer, 3.4 *μ*l 10 mM dNTPs, 0.6 *μ*l RNasin and 1 *μ*l MMLV reverse transcriptase, and 15 *μ*l of random primer/RNA mix and reconstituted to 25 *μ*l with DNase-free water (all Promega) and performed in a thermocycler at 37°C for 1 h. A reaction mix minus mRNA was prepared as a negative control. PCR was performed using 1 *μ*l cDNA and reaction reagents 5 *μ*l 5× buffer, 0.5 *μ*l 10 mM dNTP, 1.25 *μ*l 10 *μ*m forward and reverse primer (as shown in Table 1, Primer Design Ltd., Southampton, UK), 1 *μ*l 25 mM magnesium chloride, and 0.25 *μ*l Taq polymerase, reconstituted to 25 *μ*l per reaction with RNAase-free water (Promega). *β*-Actin was used as a housekeeping gene. Reactions were performed in a thermocycler, with an initial denaturing step of 94°C for 5 min, followed by 35 cycles of 1 min, 94°C denaturing step of 30 s, 55°C annealing step of 30 s, 72°C extension step, and with a final 72°C extension step for 7 min. The same conditions were applied to all primers with exception for Nanog, Oct4, CD73 and CD105, which used an annealing temperature of 52°C, and osteocalcin using annealing temperature of 62°C. Products were separated using 2% agarose gels and viewed under UV light. Images were captured using Quantity One Image Analysis Software (Bio-Rad, Hemel Hempstead, UK).

### 2.10. qRT-PCR Analysis

cDNA was generated as described above and diluted 1 : 5 with RNAase-free water. Each qRT-PCR reaction was performed using 2.5 *μ*l and 3 *μ*m primers (forward and reverse, see Table 1), 5 *μ*l diluted cDNA sample, and 10 *μ*l SYBR Green Precision qRT-PCR Master Mix (Primer Design Ltd.). All samples were analysed in triplicate in Bright White 96-well plates (Primer Design Ltd.), using an ABI Prism 7000 Sequence Detection System and ABI Prism 7000 SDS Software V1.0 (ThermoFisher Scientific). Reactions conditions were as follows: 1 cycle of 95°C for 10 min, 40 cycles of 95°C for 15 s, 55°C for 30 s, and 72°C for 30 s for all primers except for osteocalcin, where the annealing temperature was set at 62°C. For quantitative analysis, the gene expression profile was normalised to expression of *β*-actin as the housekeeping gene, using the 2^−ΔΔCt^ method for quantitative data analysis. Gene expression was presented as fold increases or decreases compared gene expression in nondifferentiating medium at day 0.

### 2.11. Assessment of Bipotential Differentiation

In order to assess the differentiation capacity of the isolated MSCs, the expression of specific differentiation markers and transcription factors was examined, both at gene and protein level. MSCs were examined at 15 PDs and 50 PDs.

#### 2.11.1. Osteogenic Induction

MSCs were seeded at 4000 cells/cm^2^ in 6-well plates for total RNA extraction and 8-well chamber slides for histological staining. Cells were cultured in CCM for 24 h, which was then replaced with osteogenic mineralising medium consisting of *α*MEM with ribonucleosides and deoxyribonuclosides, supplemented with 10% FBS, 1% antibiotics-antimycotics, 100 *μ*m L-ascorbic acid 2-phosphate, 10 nM dexamethasone, and 100 *μ*M *β*-glycerophosphate (all from Sigma-Aldrich) and further incubated in 37°C, 5% CO_2_, with medium changes every 2 to 3 days. As a negative control, cells were also cultured in CCM. At day 28, cells were fixed for 10 min with 4% paraformaldehyde and mineral calcium deposition was stained with 20 mg/ml alizarin red S (Sigma-Aldrich), pH 4.2 for 30 min, with images captured by light microscopy. mRNA expression of osteogenic markers, osteocalcin and osterix, was also examined on days 2, 7, 14, and 28 by Q-PCR, as described above.

#### 2.11.2. Adipogenic Induction

Adipogenesis was promoted using the following induction protocol. Cells were plated at 10,000 cells/cm^2^ and cultured in CCM until 90% confluent. Cells were then cultured in adipogenic induction medium (AIM; *α*MEM with ribonucleosides and deoxyribonucleosides, 10% FBS, 100 *μ*m L-ascorbic acid 2-phosphate, 1 *μ*M dexamethasone, 100 *μ*M indomethacin, 100 *μ*M 3-isobutyl-1-methylxanthine, 1% antibiotics-antimycotics (all from Sigma-Aldrich), and 37°C, 5% CO_2_ for 6 days, with media changed every 72 h). Cells were then cultured for 48 h in adipogenic maintenance medium (AMM; *α*MEM with ribonucleosides and deoxyribonucleosides, supplemented with 10% FBS, 100 *μ*m L-ascorbic acid 2-phosphate, 10 *μ*g/ml insulin, and 1% antibiotics-antimycotics) and then cultured again until day 14 in AIM. As a negative control, cells were cultured for 14 days in CCM, as described above. Intracellular lipid-rich vacuole accumulation was visualised by Oil Red O staining (500 *μ*l/well, Sigma-Aldrich; 3.5 g/l in isopropanol, mixed 3 : 2 with double-distilled water), for 10 min or the fluorescent histological stain LipidTOX™ (ThermoFisher Scientific) following the manufacturer's protocol. Digital images were captured using either a light or a fluorescence microscope, respectively. mRNA samples were collected on days 2, 7, and 14, and gene expression for adipogenic markers, adiponectin, PPAR*γ*, lipoprotein lipase, C/EBP*α*, was examined by RT-PCR and Q-PCR as described above.

### 2.12. Statistical Analysis

Data were expressed as mean ± standard error of mean (SE). Data were statistically compared using analysis of variance (ANOVA), with Tukey's post hoc test, using SPSS version 20.0 (IBM, New York, USA), with a significance level accepted at *p* < 0.05.

## 3. Results

### 3.1. Histological Analysis of Isolation Procedure

**Figure 1:**
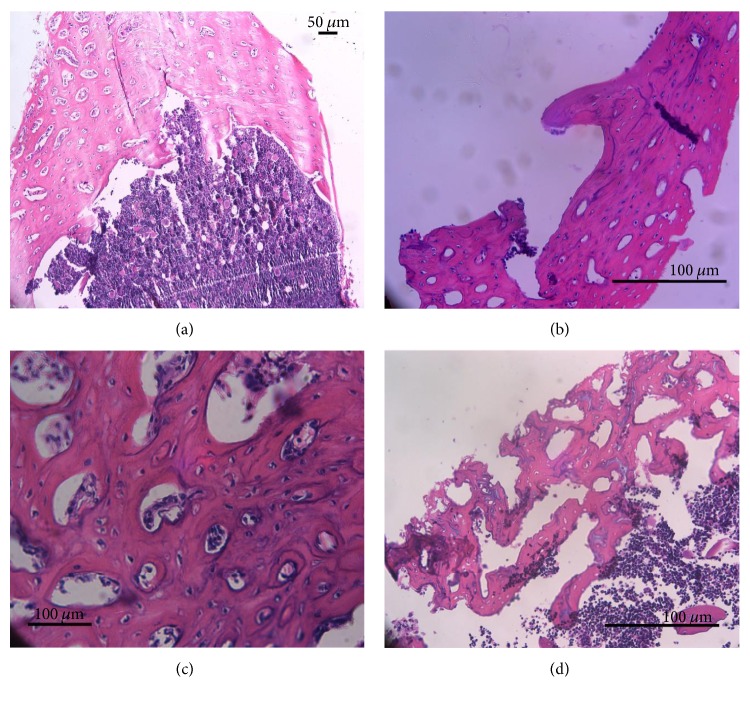
Haematoxylin and eosin staining of bone samples taken during isolation of cells from the long bones of rats. (a) Section through the femoral shaft prior to flushing of bone marrow demonstrated a large network of the Haversian and Volkmann canal system within the cortical lamellar bone. (b) Following removal of bone marrow, some cells lining the endosteal surface remain. (c) Cells are evident within the Haversian/Volkmann canal system associated with the perivascular system contained within. (d) Subsequent digestion of these bone chips with a relatively high concentration of collagenase leads to some loss of mineralised bone, opening up the canal system and associated lining cells.

Tissue samples were processed for haematoxylin and eosin staining to demonstrate cells present before flushing away of the bone marrow, after flushing away of the bone marrow, and after treatment with the collagenase. The results indicate the range of cells present in the central bone marrow cavity (Figure 1(a)), the majority of which are readily flushed away with the exception of a few remnant cells lining the endosteal surface of the bone (Figure 1(b)). The bone within the femoral shaft consisted of cortical lamellar bone, with a strong network of Haversian and Volkmann canals that were lined with perivascular-associated cells (Figure 1(c)). Treatment of this osseous tissue with the relatively high concentration of collagenase leads to some loss of the mineralised tissues, releasing cells, including perivascular MSCs and progenitor osteoblasts lining the surfaces of the Haversian and Volkmann canals (Figure 1(d)).

### 3.2. Examination of Stromal Cell Characteristics

**Figure 2:**
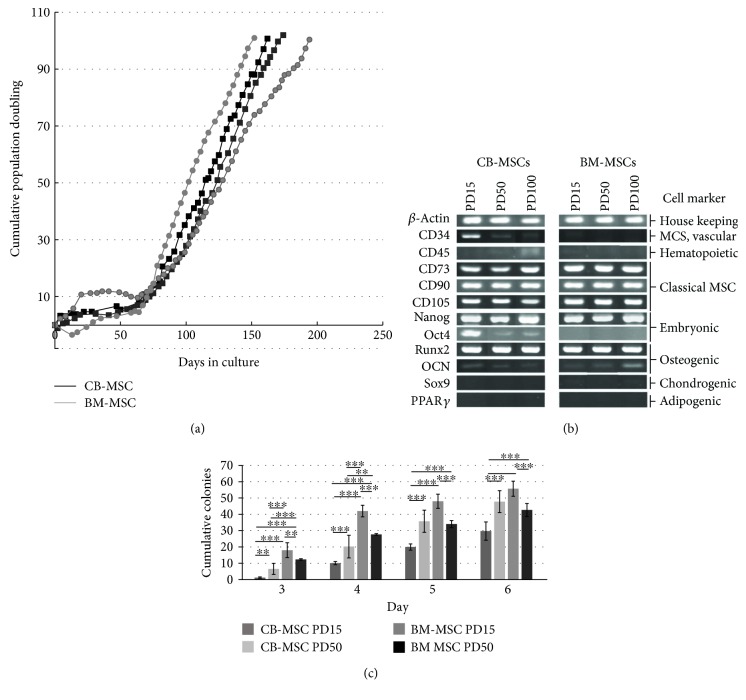
Analysis of MSC characteristics for cells isolated from compact bone and bone marrow stroma. (a) Cumulative population doubling (PDs) of CB-MSC and BM-MSC isolates, performed on two separate occasions, demonstrated high ability for self-replication after PD10, approximately 60 days in culture. (b) Both CB-MSCs and BM-MSCs expressed classical MSC markers, in addition to certain embryonic markers. Osteogenic marker, Runx2, was strongly expressed by both cell populations at all PDs examined, but were negative for chondrogenic marker, Sox9, and adipogenic marker, PPAR*γ*. CB-MSCs demonstrated the additional presence of Oct4 and CD34. (c) Colony-forming efficiency (CFEs) demonstrated lower efficiency for CB-MSC at PD15 compared to BM-MSC at PD15 and PD50. Statistically significant differences between respective populations are shown. ^∗∗∗^*p* < 0.001 and ^∗∗^*p* < 0.01.

The effects of different separation methods and niche source on the proliferative lifespan for each isolated CB-MSCs and BM-MSCs, as determined through the cumulative population doublings (PDs), are shown in Figure 2(a). Cumulative PD profiles were repeated on two different occasions and demonstrated a consistent proliferation pattern with little variation between CB-MSCs and BM-MSCs. Both populations demonstrated an initial lag phase over 60 days, followed by a rapid exponential growth reaching PD100. Both MSC populations rapidly generated a confluent monolayer from PD15, reaching 70%–80% confluence every 2-3 days; the average PDs recorded per week was 5.4 for CB-MSCs and 5.2 for BM-MSCs, respectively.

Cells from CB-MSCs and BM-MSCs, at PD 15, PD50, and PD100 positively expressed the mesenchymal progenitor cells surface antigens, CD73 (ecto-5-nucleotidase), CD90 (Thy-1), and CD105 (endoglin), and were found to be negative for the expression of CD45 (hematopoietic surface antigens) (Figure 1(b)). CB-MSCs at PD15 expressed CD34, which was lost by PD50 and absent in all BM-MSC populations. Both MSC populations maintained expression of the embryonic stem cell marker, Nanog, whilst Oct4 was only highly expressed in CB-MSCs at PD15 with faint expression at PD50 and PD100. Oct4 was absent from BM-MSCs. The expression of adipogenic differentiation marker, PPAR*γ*, and chondrogenic marker, Sox9, was negative in MSCs from both groups. Analyses also demonstrated positive expression of Runx2, along with a detectable low expression of osteocalcin in CB-MSCs and BM-MSCs at all PDs examined.

Figure 2(c) shows the CFEs for CB-MSC or BM-MSC populations at PD15 and PD50. The results indicate that CB-MSCs at PD15 have a significantly lower CFE, compared with cells at PD50 (*p* < 0.001 at days 3–6) and BM-MSCs at either PD15 or PD50. The CFE of BM-MSCs was observed to decrease as cumulative PDs increased from PD15 to PD50.

### 3.3. Characterisation of Cell Morphology and Absence of Cell Aging

**Figure 3:**
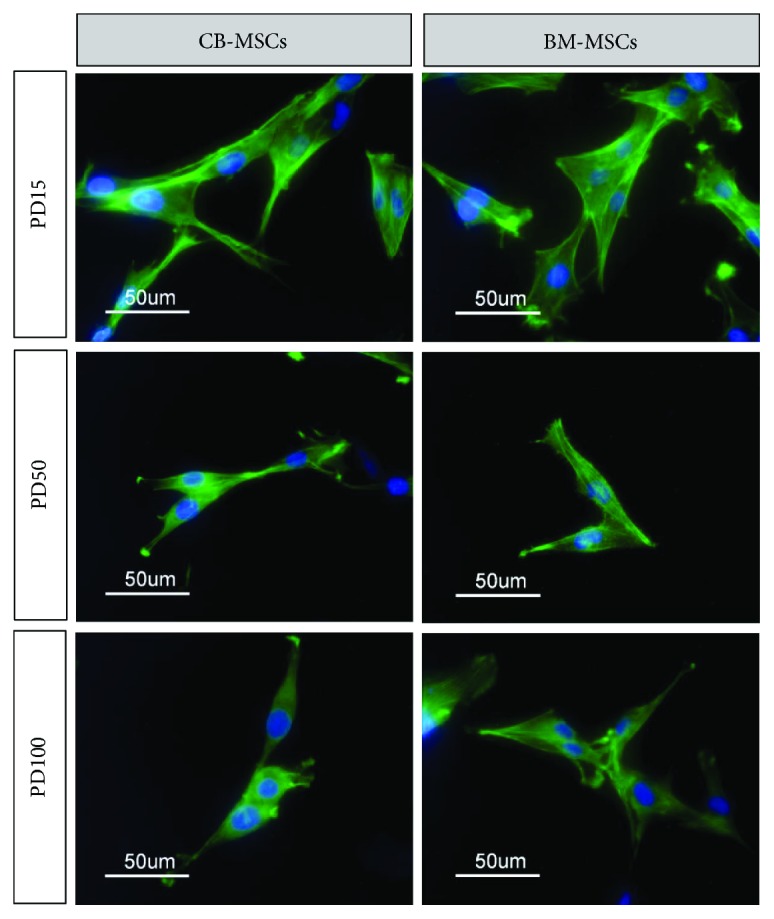
Cell morphology of CB-MSCs and BM-MSCs at PD15, PD50, and PD100 visualized following staining of cytoskeletal actin with phalloidin-FITC. At later PDs, cells were predominantly observed to adopt a more elongated fibroblastic appearance, whilst at PD15, stellate cells with increased number of cellular processes were observed.

Images presented in Figure 3 show the morphologies of cells in each MSC population at both high magnifications with actin staining. Images indicate that MSCs taken from PD15 revealed clear observation of large stellate-shaped cells, with flattened and nucleated spread body, mixed with smaller amount of spindle-shaped, fibroblastic-like morphology cells. However, at PD50, the smaller cells with a more fibroblastic-like appearance were the predominant cell type present, with an organised pattern of actin cytoskeleton, formed centrally within spindle-shaped cells in the culture. At PD100, both CB-MSC and BM-MSC populations demonstrated the presence of small stellate-like with short processes.

**Figure 4:**
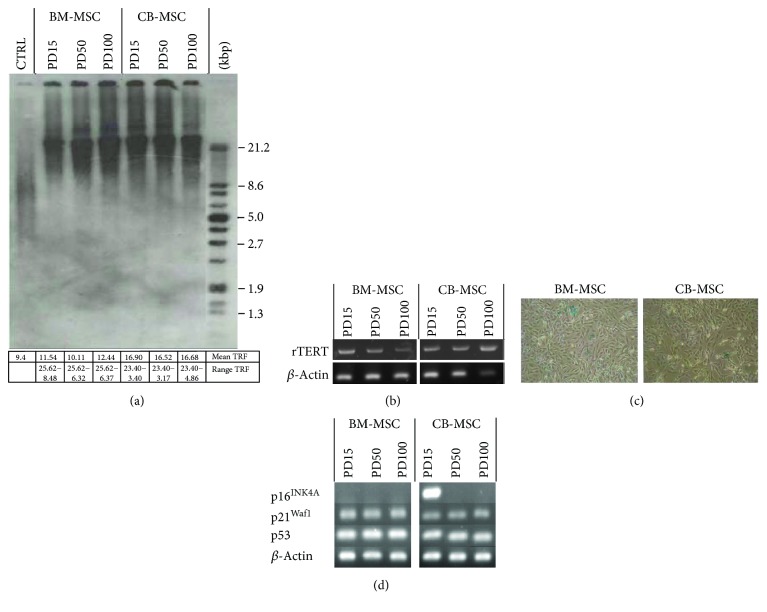
Effect of continuous cell expansion on (a) telomere length analysis for CB-MSC and BM-MSC populations at cited PDs, by Southern blotting. Average telomere lengths were calculated from 3 blots, and averages and range of TRF values obtained are indicated in the associated table. Right-hand lane represents the separated DIG-labelled telomere length standard. (b) Expression of telomerase catalytic subunit, rTERT, was assessed by RT-PCR. Continued expression of rTERT at PD100 is consistent with minimal change in telomere length following expanded culture. (c) Staining of cell populations at PD100 with SA-*β*-galactosidase indicated the absence of senescent cells. (d) mRNA expression of cell cycle proteins regulating arrest in CM-MSCs and BM-MSCs was assessed by RT-PCR. p16^INK4A^ was only detected in CB-MSCs at PD15 and was lost with subsequent culture. Continued expression of p21^Waf1^ and p53 was observed.

During this extended culture, CB-MSCs or BM-MSCs showed little signs of cellular senescence (Figure 4). Analysis of telomere lengths indicated little shortening during extended culture (Figure 4(a)), which correlated with the continued expression of rTERT (Figure 4(c)). BM-MSCs had slightly longer telomere lengths, ranging from approximately 25.6 to 6.3 kbp, compared to CB-MSCs which ranged from 23.4 to 3.1 kbp, but these represent average values from 3 separate blots and were deemed to be statistically nonsignificant. Further analyses indicated that cells were nonsenescent, which was confirmed by <2% of cells per 30 *μ*m^2^ staining positive for SA-*β*-galactosidase (Figure 4(d)) and cells still being able to achieve a doubling rate > 4.9 PD/week (Figure 2(a)).

Analysis of genes associated with cell cycle regulation (Figure 4(d)) showed that all cell populations expressed the tumour suppressor gene, p53, and downstream gene, p21^Waf1^ (regulator of cell cycle progression at G1 and S phase). However, another recognized tumour suppressor gene, p16^INK4A^, was only expressed in CB-MSCs at PD15, which was lost by PD50.

### 3.4. Osteogenic and Adipogenic Differentiation of MSCs

**Figure 5:**
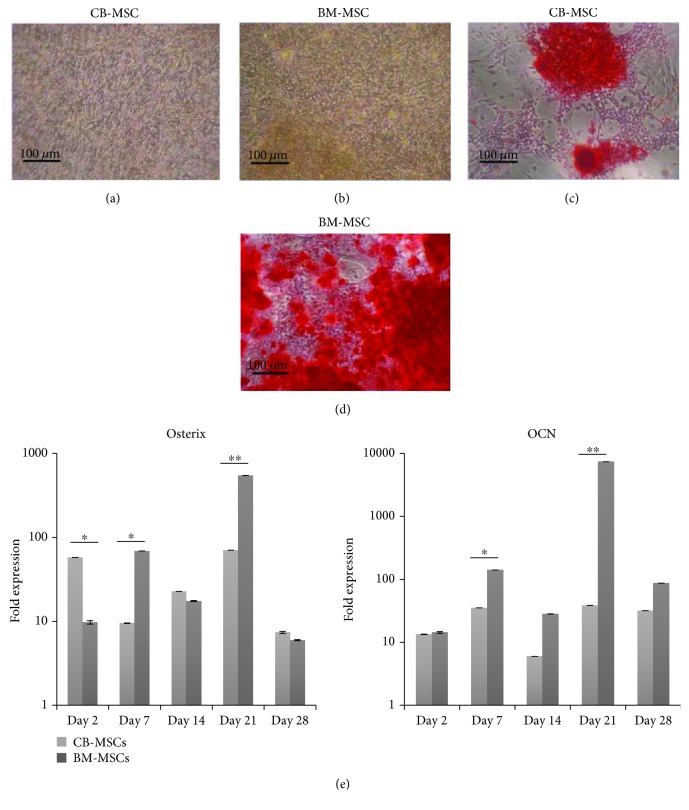
Osteogenic differentiation of CB-MSCs and BM-MSCs at PD50. Cells were cultured for 28 days in the absence (a and b) or presence (c and d) of osteoinductive media and mineral deposition visualised by alizarin red staining. At PD50, osteogenic potency was observed to be higher for BM-MSCs, a result which is supported by (e) gene expression profiles for osteogenic markers by CB-MSCs and BM-MSCs. Values were normalised against the expression of *β*-actin and presented as the fold increase compared to expression value obtained for the respective cell population in nondifferentiating media, at day 0. Each bar represents mean fold change ± SD, *n* = 3. ^∗^*p* < 0.05 and ^∗∗^*p* < 0.01.

At PD50, following 28 days culture in osteogenic medium, both MSC populations demonstrated distinct formation of mineralised bone nodules that stained positive with alizarin red (Figures 5(c) and 5(d)). Staining was greater in BM-MSCs. These results were confirmed by the analysis of gene expression for osteoblast-specific markers, osterix and osteocalcin (OCN) by qPCR (Figure 5(e); presented as fold differences compared to before and after treatment with the inductive medium). Analyses indicated that in the presence of osteoinductive media, both CB-MSC and BM-MSCs showed considerable fold-change increases in these osteogenic genes during induction, with a significantly greater fold increase observed in BM-MSCs noted at days 7 and 21 (*p* < 0.01). CB-MSCs indicated higher fold-change increases in osterix at day 2, but this high expression was not sustained as cultures continued in osteoinductive media.

**Figure 6:**
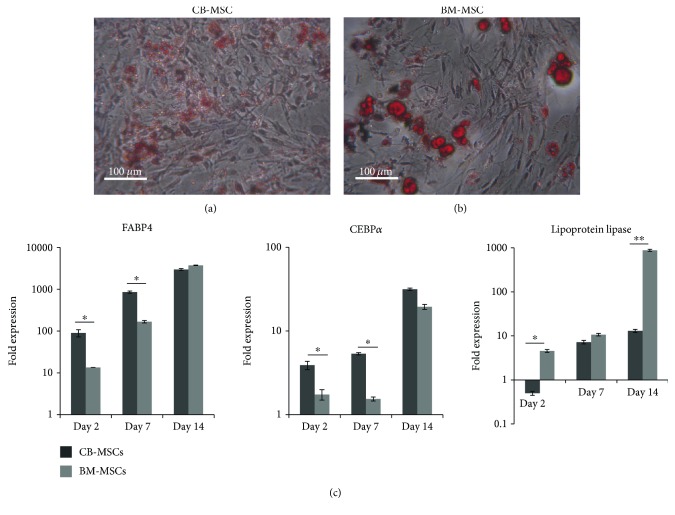
Adipogenic induction of CB-MSCs and BM-MSCs at PD50. (a) CB-MSCs and (b) BM-MSCs were cultured in adipogenic medium for 14 days and stained with Oil Red O to detect neutral lipid droplet formation within the cultured cells. Potency was greater for BM-MSCs with results correlating with (c) gene expression of adipogenic markers in MSCs at PD50 quantified by qPCR. Values were normalised to the expression of *β*-actin, and expression following 14 days in adipogenic media is presented as a fold increase or decrease compared to levels in cells cultured in basal media. Each bar represents the mean fold change ± SD. *n* = 3, ^∗^*p* < 0.05, and ^∗∗^*p* < 0.01.

**Figure 7:**
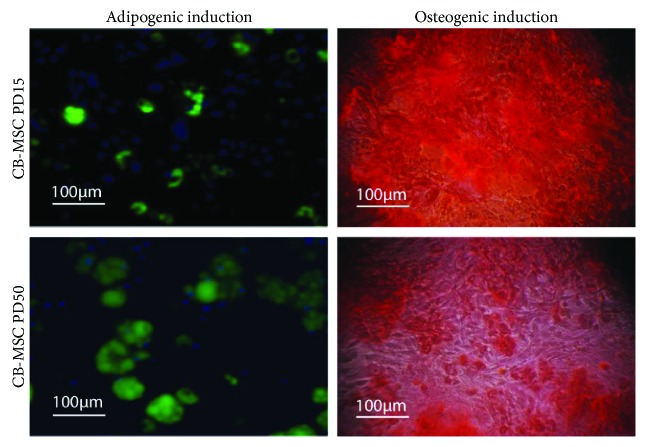
Comparison of CB-MSCs at PD15 and PD50 for adipogenic and osteogenic induction. Cells at PD15 demonstrated a greater potency for osteogenic induction and reduced adipogenic induction compared to cells cultured to PD50.

Adipogenic differentiation was also successfully induced in both CB-MSC and BM-MSCs, with Oil Red O staining indicating the formation of larger and clearer lipid droplets in the differentiated BM-MSCs (Figures 6(a) and 6(b)). These observations correlated with the higher gene expression of lipoprotein lipase in BM-MSCs, with roles proposed to be involved in fatty acid uptake and storage (Figure 6(c)). However, qPCR also demonstrated the earlier induction of adipogenic master gene, C/EBP4, and downstream gene, FABP4, within the CB-MSC cell population (Figure 6(c)). The adipogenic and osteogenic potency of CB-MSC at PD15 and PD50 was also examined using staining techniques for mineral deposition using alizarin red and formation of lipid droplets using the fluorescent dye, LipidTOX (Figure 7). At PD50, CB-MSCs demonstrated an increased potential for adipogenic induction and a reduced potential for osteogenic induction, compared with CB-MSCs at PD15.

## 4. Discussion

The present study presents an original comparative analysis pertaining to the isolation, expansion, and characterisation of heterogeneous MSC populations potentially derived from the cell niches associated with bone marrow stroma (achieved via preferential fibronectin adherence plating techniques; BM-MSCs), and with the endosteal surface of rat bone chip explants (CB-MSCs). Regardless of cell origin and isolation technique, all purified MSC populations were found to be positive for selected MSC markers, CD105, CD90, and CD73, indicating that they were of mesenchyme origin and meeting the suggested requirements of the International Society of Cell Therapy (ISCT) position statement [13]. These populations were negative for CD45, indicating that the isolated population was successfully separated from hematopoietic stem cells that are present in high numbers in the bone marrow stroma and associated with the endosteal niche [8] which would interfere with a full characterisation of MSCs. Both isolation techniques were successful in the isolation of multipotent MSCs which were capable of sustaining long-term *in vitro* expansion up to PD100. Analysis of cells at PD50 indicated that both populations maintained MSCs that were bipotential for osteoblastic and adipogenic lineage differentiation. For both CB-MSC and BM-MSC cell populations, cell doubling only significantly increased when the cells had achieved approximately PD10. Similar lag periods in high-proliferative activity have been reported previously [20] and possibly reflect the expected low numbers of highly proliferative MSCs expected in these isolations, which subsequently expand to become established within the heterogeneous MSC population, and/or cells adapting to new culture conditions which differ to the *in vivo* niche environment. For this reason, all analyses were performed when cells were rapidly proliferating at PD15 and beyond—a time period when cells are also more likely to be utilized for *in vitro* assay model systems or cell therapies.

Within this study, cells at PD15 for both CB-MSC and BM-MSC demonstrated a heterogeneous morphology with both small, elongated bipolar cells and larger polygonal cells present. This is consistent with previous studies examining stromal cell precursors isolated from the bone marrow, which have continued to identify that the smaller fibroblastic-like cells of 60–100 *μ*m are also actively proliferating whilst the larger polygonal cells proliferate more slowly [12]. Of note, the larger cells appear to be lost by PD50, which may be due to their slower proliferation rate compared with the smaller faster dividing cells which thus dominate the culture. The smaller cells may thus represent cells which conform to transit-amplifying cells with greater multipotentiality, whilst the larger cells may represent either more differentiated, lineage-committed cells or larger aging, senescent cells. The latter is the less likely scenario since telomere lengths remained consistently long and unchanged during culture expansion (25–8 kbp), an observation supported by the detection of high levels of rTERT expression. Further analysis of cells at PD50 indicated few senescent cells staining positive for SA-*β*-galactosidase, suggesting that these cells may be present albeit in low numbers, although more analysis may be necessary to verify this conclusion. The presence of preosteoblastic stromal cells along with nestin-positive MSC has been described within two important niches within the bone marrow stroma, namely, the perivascular niche and the endosteal niche, where they are important in supporting hematopoietic stem cells, in addition to providing an important contribution to bone remodeling and repair activities [8]. Their presence thus contributes to the heterogeneous nature of mesenchymal stem cells, which are evident in either isolations from bone marrow (BM-MSCs) or as a result of explants following collagenase digestion that remove all but cells associated with the endosteal niche (CM-MSCs). CM-MSCs are likely to additionally contain perivascular MSCs, preosteoblasts, and bone-lining cells that also reside within the Haversian and Volkmann canal system, which takes a vascular supply from the periosteum into the bone, in addition to supporting the osteocyte network and physiological bone remodeling.

Both BM-MSC and CB-MSC populations demonstrated a consistent expression of the embryonic stem cell marker, Nanog, at all stages of culture. Nanog has been proposed a role in supporting the proliferation of adult MSCs in the transition from *in vivo* quiescence to adaption expansive growth *in vitro* [24, 25]. However, within this study, we also identified MSC populations within CB-MSC PD15 cell population which were CD34^+^ and Oct4^+^. A CD34^+^ MSC subpopulation has been described within the bone marrow, proposed to be intimately associated with the vasculature [26], and thus may relate to a quiescent arterial niche recently described to be present in the endosteal region [27, 28]. Expression of CD34 appears to be influenced by the surrounding environment and it has been documented that MSC expression of CD34 can change from positive to negative and vice versa, which may account for the loss of expression in the CB-MSC cultures beyond PD15 [25]. Oct4 has proposed roles in maintaining pluripotency and is used in the generation of induced pluripotent cells from adult MSCs [24, 29] and, of note to this present study, “induced MSCs” from CD34^+^ cells isolated from adult peripheral blood [30]. Collectively, the identification of these MSC markers, Nanog, CD34, and Oct4, within our CB-MSC populations would suggest the presence of an immature MSC subpopulation maintained in an undifferentiated state, capable of propagation for multiple passages at least beyond PD15, which is not identifiable in MSC populations derived from the bone marrow stroma.

Irrespective of PDs, all isolated CB-MSCs and BM-MSCs in the present study showed positive expression of Runx2 during expansion culture in basal medium. Although its role has not been fully delineated, Runx2 and osterix are required as essential to promote osteoblast differentiation at an early stage of induction, but inhibited terminal osteoblast differentiation [31]. Runx2, with Sox5, Sox6, and Sox9, is essential for the terminal differentiation of chondrocytes [31]. Within the study presented herein, we failed to detect Sox9 and detected low levels of osterix, suggesting that both the CB-MSC and BM-MSC heterogeneous populations also contained osteoprogenitor and preosteoblasts, cells which studies have shown are still capable of extensive replication [32]. This commitment to the osteoblast lineage, however, is not expected to negate a contribution to the bipotentiality seen with both cell populations. Studies have indicated that the transcription factor, PPAR*γ*, can induce the transdifferentiation of Runx2 expressing osteoblastic MC3T3-E1 cells into mature adipocytes [33].

Within their respective heterogeneous populations, both CB-MSC and BM-MSC populations demonstrated similar population doubling profiles, achieving between 4 and 5 PDs per week. Within each MSC population, the expression of the cell cycling genes regulating replicative capacity was also examined. p21^waf1/CIP1^ and p53 can act in dependent and interdependent ways to arrest cell cycle progression at G1 and S phases, acting as tumor suppressors to regulate cell proliferation to rates that provide genomic fidelity during MSC division [34]. p53 is also a negative regulator in the formation of preosteoblasts and preadipocytes [35], and loss of p53 leads to osteosarcoma development [36]. The expression of p21^waf1/CIP1^ and p53, therefore, regulates the steady expansion of MSCs in an undifferentiated state, and their presence in both populations suggests the presence of such MSC populations. In addition, CB-MSCs at PD15 demonstrated the additional presence of p16^INK4A^, which also has a proposed role in reducing stem cell proliferation [37, 38]. It has also been suggested as a key marker of stromal cell and somatic cell senescence [39] and may indicate the isolation of senescent cell subpopulations within CB-MSC isolates, although telomere measurements suggested little reduction in lengths. It is noteworthy, however, that protein expression of p16^INK4A^ and p21^waf1/CIP1^ has both been detected in fully differentiated osteoblasts derived from MC3T3 cells and calvarial explants, where it has been proposed that they are involved in cell growth arrest in this scenario [40]. It is thus tempting to speculate that the presence of p16^INK4A^ within the CB-MSC population at PD15 represents a subpopulation of postmitotic differentiated osteoblast cells, possibly derived from the bone-lining cells within the endosteal environment. To test this hypothesis, we examined the ability of cells within the respective populations to form colonies from a single cell, correlated to their bipotential potency. The present study clearly demonstrated that CFEs for the CB-MSC population at PD15 were significantly lower compared to CFEs for CB-MSCs at PD50 and BM-MSCs at either PD15 or PD50. However, the CB-MSC population at PD15 demonstrated increased staining for the deposition of mineral containing bone nodules and reduced potential to form adipocytes, when compared to CB-MSCs at PD50, thus supporting the presence of more mature osteoblasts in the heterogeneous population at PD15 of the CB-MSC population, which are subsequently lost by PD50.

In the present study, we compared CFEs and bipotentiality to form osteoblasts and adipocytes by CB-MSC and BM-MSCs at PD50, a time when both cell populations showed similar gene expression profiles for MSC markers, CD73, CD90, and CD105, Nanog, the osteogenic marker Runx2, and cell cycle proteins, p21^waf1/CIP1^, p53 (CD34, Oct4, and p16^INK4A^ positivity was not detected). At this time point, BM-MSCs had a greater potential for adipogenic and osteogenic differentiation, compared to CB-MSCs. Both populations demonstrated a marked ability to form clonal colonies. These results would suggest that the bone marrow population contained greater numbers of committed preosteoblasts, which are still capable, under the correct signalling environment, to form adipocytes [33, 41]. In contrast, considering CB-MSCs at PD50, a more immature MSC population now predominated. As these CB-MSC cultures were continued under osteogenic conditions, increased deposition of mineralizing bone nodules was observed, suggesting the presence of cells with osteogenic potential, which took longer to achieve a fully differentiated osteoblast capable of synthesizing mature bone.

## 5. Conclusion

The study has compared two isolation techniques which have yielded two heterogeneous MSC populations whose characteristics vary according to the niche source of the cells. The value of the data is twofold. The study offers data to provide an informed consideration for which isolation technique to use in selecting an *in vitro* model. The protocols have been successful in isolating MSCs from the compact bone niche, a MSC population that is more lineage restricted for osteogenesis. Such cells may be proposed to be the first responders during mineralized tissue repair, with the immature MSCs subsequently proliferating to replace the former cell population [42]. Thus, isolation of primary cells from bone explants offers MSCs which may be more appropriate when wishing to assess the efficacy of osteogenic therapeutics, where data obtained may be more relevant for the study of bone repair processes. Conversely, BM-MSCs may be more appropriate when assessments of tripotentiality are required and could find relevance in the study of a range of tissue engineering applications outside bone regeneration. In addition, the data provides an important initial characterisation to justify studies that further characterise the MSC populations within human models. It is of note that the gold standard for bone regeneration is autologous bone grafts that contain an endosteal niche and hence a potential benefit from a MSC population that is lineage-restricted in terms of osteogenic differentiation. Further investigation of these lineage-restricted MSCs may thus be of benefit in providing a more defined cell population for stem cell-based therapies.

## Figures and Tables

**Table 1:** Primer sequences for RT-PCR and qPCR analysis.

Gene	Primer sequence	Length (bp)	Application
CD73	F: 5′ TCCCGCGGCTGCTACGGCACCCAAGTG-3′ R: 5^'^-ACCTTGGTGAAGAGCCGGGCCACGCCG-3′	204	RT-PCR
CD90	F: 5′-CCTGACCCGAGAGAAGAA-3′ R: 5′-TGAAGTTGGCTAGAGTAAGGA-3′	125	RT-PCR
CD105	F: 5′-ACATGGTGCCCACACCCGCAGCTGGCA-3′ R: 5′-CACTGCCACCACGGGCTCCCGCTTGCT-3′	263	RT-PCR
CD45	F: 5′-AGCAATACCAGTTCCTCTATGA-3′ R: 5′-TCCGTCCACTTCGTTATGA-3′	113	RT-PCR
CD34	F: 5′-GTCACACTGCCTACTACTTC-3′ R: 5′-TCCTCGGATTCCTGAACAT-3′	210	RT-PCR
Nanog	F: 5′-GGGGATTCCTCGCCGATGCCTGCCGTT-3′ R: 5′-GGGATACTCCACCGGCGCTGAGCCCTT-3′	477	RT-PCR
Oct4	F: 5′-GCCCACCTTCCCCA TGGCTGGACACCT-3′ R: 5′-GCAGGGCCTCGAAGCGGCAGA TGGTTG-3′	563	RT-PCR
Runx2	F: 5′-CCAGATGGGACTGTGGTTACC-3′ R: 5′-ACTTGGTGCAGAGTTCAGGG-3′	381	RT-PCR
OCN	F: 5′-ACAGACAAGTCCCACACAGCAACT-3′ R: 5′-CCTGCTTGGACA TGAAGGCTTTGT-3′	161	RT-PCR
PPAR*γ*	F: 5′-GGAAAGACAACAGACAAA TCAC-3′ R: 5′-GAACTTCACAGCAAACTCAAAC-3′	408	RT-PCR
Sox9	F: 5′-CCCTTCAACCTCCCACACTACAGC-3′ R: 5′-TGTGTAGACGGGTTGTTCCCAGTG-3′	249	RT-PCR
rTERT	F: 5′-GACATGGAGAACAAGCTGTTTGC-3′ R: 5′-ACAGGGAAGTTCACCACTGTC-3′	185	RT-PCR
p53	F: 5′-ACAGCGTGGTGGTACCGTAT-3′ R: 5′-GGAGCTGTTGCACATGTACT-3′	83	RT-PCR
p21^waf1^	F: 5′-TCTTGCACTCTGGTGTCTCA-3′ R: 5′-GGGCTTTCTCTTGCAGAA-3′	147	RT-PCR
p16^INK4A^	F: 5′-TGCAGATAGACTAGCCAGGGC-3′ R: 5′-CTCGCAGTTCGAATCTGCAC-3′	184	RT-PCR
*β*-Actin	F: 5′-TGAAGATCAAGATCATTGCTCCTCC-3′ R: 5′-CTAGAAGCATTTGCGGTGGACGATG-3′	155	RT-PCR
OSX	F: 5′-GCTTTTCTGTGGCAAGAGGTTC-3′ R: 5′-CTGATGTTTGCTCAAGTGGTCG-3′	200	qPCR
OCN	F: 5′-ACAGACAAGTCCCACACAGCAACT-3′ R: 5′-CCTGCTTGGACATGAAGGCTTTGT-3′	161	qPCR
FABP4	F: 5′-GGAATTCGATGAAATCACCCC-3′ R: 5′-TGGTCGACTTTCCATCCCACT-3′	104	qPCR
C/EBP*α*	F: 5′-GGGAGAACTCTAACTCCCCCAT-3′ R: 5′-CTCTGGAGGTGGCTGCTCATC-3′	82	qPCR
LPL	F: 5′-AGGTCAGAGCCAAGAGAAGCA-3′ R: 5′-GGAGTAGGTTTTATTTGTGGCG-3′	215	qPCR
*β*-Actin	F: 5′-GGGTCGAGTCCGCGTCCAC-3′ R: 5′-CGACGAGCGCAGCGATATC-3′	108	qPCR
